# Efficacy of buccal pad fat as a new approach in the treatment of gingival recession: a systematic review

**DOI:** 10.1186/s12903-024-04519-9

**Published:** 2024-07-09

**Authors:** Reham Abdel-Fatah, Wafaa Saleh, Hesham El-Sharkawy

**Affiliations:** Oral Medicine, Periodontology, Diagnosis and Oral Radiology Department, Faculty of Dentistry, Mansoura University, Algomhoria St, Mansoura, Aldakhlia 35516 Egypt

**Keywords:** Buccal pad fat, Miller’s classification, Gingival recession, Treatment

## Abstract

**Objective:**

This systematic review evaluates the efficacy of buccal pad fat (BPF) as an autologous graft in the treatment of gingival recession (GR). Thus, the research question explores if the BPF can serve as a viable alternative to the gold standard connective tissue graft.

**Materials and methods:**

Only seven studies met the inclusion criteria were critically appraised including the randomized controlled clinical trials, and case series. The inclusion criteria were systemically healthy individuals in the age range (18–65 years old) with Miller’s classification of GR either class I, II, III, or IV while exclusion criteria were patients with poor oral hygiene, pregnant and lactating patients, teeth with caries, any prior surgery in the relevant regions, and use of medications.

**Results:**

The review included 117 patients with 136 GR defects. The age of participants ranges from 20 to 65 years old with the higher percentage of root coverage (%RC) at 6 months in the pedicled BPF group which was 89.30%while the lowest (%RC) at 6 months in the same group was 46.78%. The BPF group’s width of keratinized gingiva (WKG) values indicate a notable improvement, suggesting a positive impact on WKG compared to the control group.

**Conclusions:**

BPF can be considered as a promising graft to augment gingival tissues at different sites in the oral cavity with different Miller’s classes of GR providing a new era in GR treatment.

**Supplementary Information:**

The online version contains supplementary material available at 10.1186/s12903-024-04519-9.

## Introduction

Gingival recession (GR) is defined as root exposure which results from the apical migration of the gingival margin with subsequent dentin hypersensitivity, root caries, and compromised esthetics [1, 2]. Various factors contribute to GR, including plaque-induced gingival inflammation, faulty tooth brushing, malocclusion, orthodontic treatment, and high frenum pull. Furthermore, predisposing factors like thin gingival phenotype and a positive history of progressive GR have been reported to increase its incidence [3–5].

Interestingly, numerous GR treatment modalities have been introduced during the last decades such as different flap techniques including the coronally advanced flap (CAF), laterally positioned flap, various tunneling techniques either alone or in combination with several grafting materials such as subepithelial connective tissue graft (SCTG), acellular dermal matrix, collagen substitutes and different biologics as enamel matrix derivatives, platelet–rich fibrin (PRF), and hyaluronic acid. Moreover, emerging treatment modalities have also evolved during the last years, among which the microsurgical techniques, novel grafting materials such as amniotic membrane, chorion membrane, and more recently buccal pad fat (BPF) graft [6–9].

BPF is an encapsulated fat mass located bilaterally in the cheek mucosa and surrounded by the buccinator muscle, the masseter muscle, and the two zygomatic muscles with its inferior portion in the buccal space. It is composed of a central body with four extensions being buccal, pterygoid, pterygopalatine, and temporal one [10]. BPF contains abundant neural-crest-derived stem cells, and blood vessels [11]. It maintains an average volume of about 9.6 ml [12] which is being fairly constant in all individuals even the cachectic people [13, 14]. BPF is composed of tissue lipids which improve intermuscular movement and exhibit lower susceptibility to lipolysis compared to other body fats [15].

Besides, BPF resists infection, and necrosis, and can keratinize within 3–6 weeks [15–17]. Interestingly, the stem cells derived from the BPF experimentally exhibit an earlier expansion rate with increased osteogenic and angiogenic cell surface markers, compared to other body adipose tissue-derived stem cells such as the hip and abdomen [18].

Free autologous BPF has been utilized for more than a century in both esthetic and reconstructive surgeries due to its well-established clinical characteristics, healing properties, and reliable outcomes [19]. Orally, the BPF was first introduced by Egyedi in the closure procedures of oro-nasal/antral communications and has since been employed in reconstructing various soft tissues following both traumatic and malignant lesions such as soft palate, hard palate, buccal mucosa, retromolar area, and anterior tonsillar pillar [20, 21].

BPF can be utilized as a pedicled graft, offering proximity to the donor site, a rich vascular supply from various arteries, consistent weight among individuals, simplicity, ease of harvesting, possibility to mobilize/adapt, lower infection rate, and keratinizing properties. This encourages its usage in oral reconstructions and recently as a grafting material in severe GR treatment of maxillary molars [22–24]. However, fibrosis is the main healing mechanism of autologous BPF as the exposed fat tissues become yellowish or whitish after 3 days and then become reddish after 1 week due to immature granulation tissue formation [25, 26].

The introduction of the free BPF (FBPF) graft in 2011 represented a clinical innovation, with subsequent studies evaluating its reliability in various intraoral surgical reconstructions, including GR treatment [27, 28]. The merits of FBPF include donor site accessibility to the surgeon, minimal site morbidity, and minimal patient discomfort. More importantly, surgical procedures do not impact the appearance or structure of the donor site [29].

While literature describes the GR treatment using pedicled BPF and, more recently, FBPF with satisfactory results in clinical attachment level (CAL), root coverage percentage (RC %), and keratinized tissue gain [22, 27, 30], there is a notable absence of systematic reviews evaluating the overall effectiveness of BPF in treating GR. Therefore, this systematic review aims to assess the different BPF techniques used for GR treatment.

## Materials and methods

### Protocol registration

This systematic review is designated by the Preferred Reporting Items for Systematic Reviews and Meta-Analysis (PRISMA) guidelines with the study protocol design following the Cochrane Handbook for Systematic Reviews of Interventions that is a recently updated in 2023 [31]. The study protocol was registered in the PROSPERO database under the following registration number (CRD42023485492).

### Focused PICOS questions

The focused PICOS questions for this systematic review are illustrated below.

#### Population (P):

Patients with GR (Class I, II, III or IV Miller’s classification of GR).

#### Intervention (I):

BPF graft (either free or pedicled) with CAF or vestibular incision subperiosteal tunnel access (VISTA) technique.

#### **Comparisons (C**):

Comparison performed with other biomaterials, PRF, SCTG, or Emdogain.

#### Outcomes (O):

Recession depth (RD), recession width (RW), width of keratinized gingiva (WKG), thickness of keratinized gingiva (TKG), probing depth (PD), CAL, and %RC.

#### Study design (S):

Studies included (Randomized clinical trials (RCTs), non-randomized clinical trials, single arm trials and case series).

#### Database searching:

PubMed, Cochrane, Google scholar, Web of science, Scopus, and EMBASE.

### Search strategy

The electronic database search was conducted until December 30, 2023. All studies investigating the use of BPF as a graft material in the treatment of GR included both free and pedicled grafts. Furthermore, this review encompasses all published articles on BPF in GR treatment, including RCTs and case series with representative sample size. This comprehensive approach revealed only a limited number of studies identified in the initial review on this research topic.

### Inclusion criteria

We included systemically healthy (medically free) individuals in the age range (18–65 years old) with Miller’s classification of GR either class I, II, III, or IV [32].

The selected articles met specific predefined criteria:

- Studies published in English.
- Randomized clinical trials (RCTs), observational studies and case series.
- Studies that reported clinical outcomes of interest.

### Exclusion criteria

The selected articles excluded patients with poor oral hygiene, pregnant and lactating patients, teeth with caries, or restorations, any prior periodontal surgery in the relevant regions, and use of any kind of medications that could interfere with the health of gingival or periodontal tissue. Studies with inadequate follow-up periods or incomplete data regarding gingival recession treatment outcomes were excluded. Additionally, case reports and case series with small sample sizes (less than 10 cases) were excluded from this systematic review.

### Article selection process

Two independent reviewers (R.A and W.S) performed the initial screening of the searched databases including (PubMed, Cochrane Central, Google scholar, Web of science, Scopus, and EMBASE) to select the eligible articles. A comprehensive search strategy included terms such as buccal pad fat, buccal fat pad, gingival recession, gum recession, periodontal recession, and gingival recession treatment. Additionally, relevant terms encompassing BPF grafting, such as buccal pad fat grafting, buccal pad fat transplantation, and soft tissue augmentation, were included. Other terms such as connective tissue graft, coronally advanced flap, root coverage procedures, mucogingival surgery, and periodontal plastic surgery were considered.

**Fig. 1:**
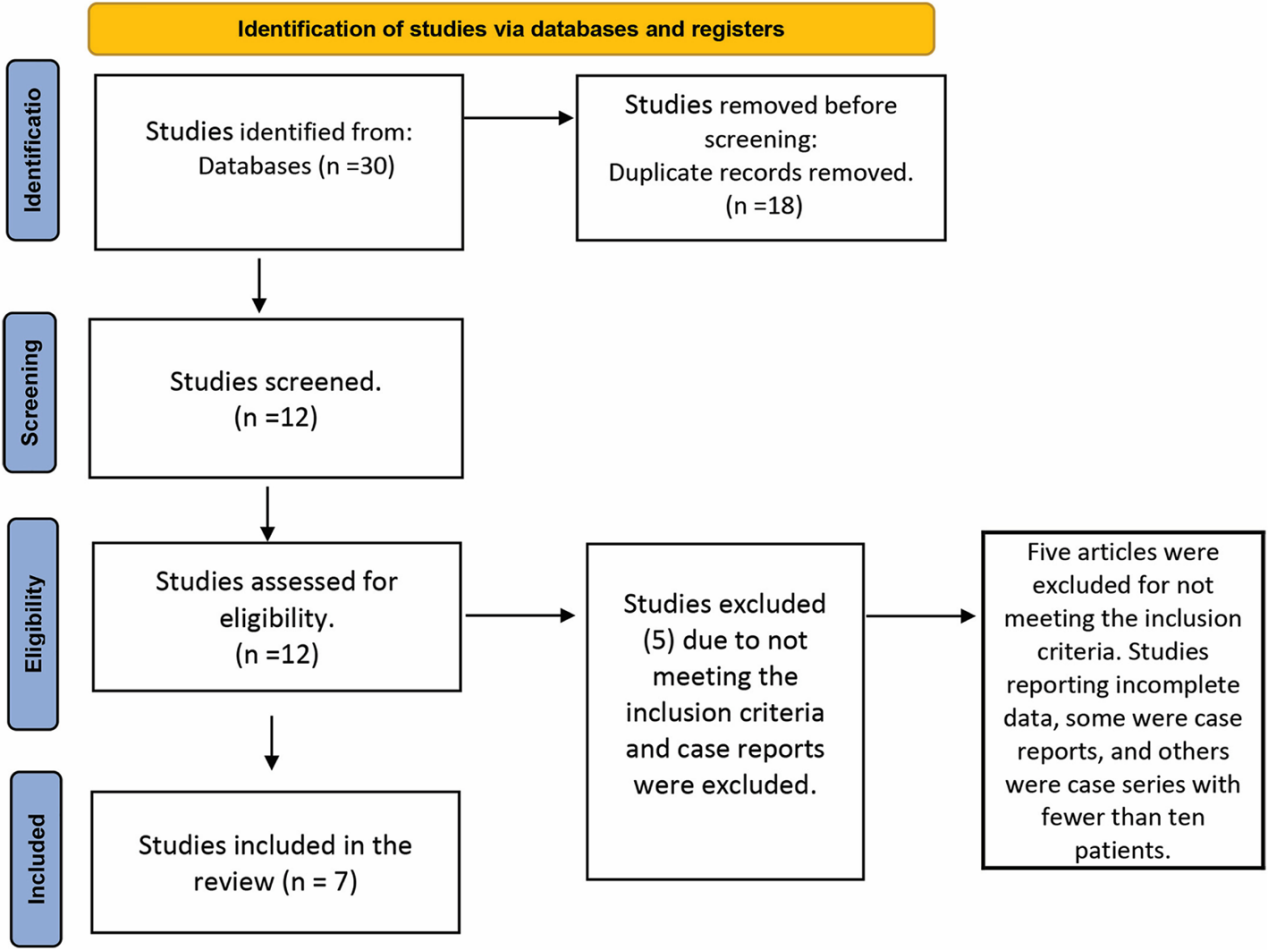
Study flow chart

Subsequently, the full texts of the chosen articles underwent scrutiny, encompassing the removal of any duplications. Finally, the selected articles were reviewed for full-text assessment and final selection. Any disagreements between the two reviewers were resolved by open discussion, and if no agreement could be reached, a third author (H.E.) was consulted. Studies failing to align with the previously specified inclusion criteria (as depicted in Fig. 1) were also excluded from the analysis.

### Data extraction

The data were extracted in duplicate by two reviewers (R.A and W.S.) independently. The following items were recorded: (1) Study design; (2) Participants’ characteristics (i.e. number, age, sex, and smoking status); (3) Recession defect characteristics (i.e. number of defects in each control and test group, defect site, defect classification, surgical techniques used in each group, and follow-up duration); (4) Main outcomes of the study (shown in Table 1).

**Table 1:** General characteristics of the selected studies

Study ID	Study Design	No. of participants	Age range (in years)	Sex (M: Male F: Female	Smoking status	No. of recession defects per group		Recession sites	Class of gingival recession	Surgical techniques		Follow up duration in months(m)	Main outcomes
						Study group	Control group			Study group.	Control group.		
**1.Deliberador, 2015** [27]	Clinical trial	12	21–65	M:4, F:8	Excluded	12	12	Maxillary premolars & canines	class I &II miller	CAF &NPBPF	CAF & SCTG	1,3,6 m	Non pedicled BPF (NPBPF) can be used in class I and II GR treatment with comparable results to SCTG, but further studies are required to confirm BPF graft efficacy.
**2. El-Haddad, 2017** [33]	Clinical Case series	10	20–50	NR	Excluded	5	5	Maxillary first molars	class IV Miller	PBPF	PBPF & Emdogain	3,6, 24 m	Pedicled BPF can be used in the treatment of severe maxillary posterior teeth GR to preserve the hopeless molar and augment the keratinized gingival tissue.
**3.Deepa&Kumar 2018** [34]	Clinical study	10	35–55	M:6, F:4	Excluded	10	NA	Maxillary first & second molars	class II & III Miller	PBPF	None	6 m	Pedicled BPF (PBPF) can be used as an alternative treatment modality in the severe maxillary posterior GR.
**4.Kablan, 2018** [28]	Clinical study	10	31–45	M:4, F:6	NR	17	NA	Maxillary & mandibular teeth	NR	CAF & NPBPF	None	12 m	NPBPF serves as an excellent option for the treatment of severe GR in both jaws with the long-term stability of the obtained results.
**5.Khalil, 2019** [36]	Clinical study	20	25–48	NR	Excluded	10	10	NR	class I & II Miller	VISTA & NPBPF	VISTA & SCTG	3,6 m	NPBPF can be used in multiple class I and II GR treatments with the VISTA technique with comparable results to SCTG, and little donor site morbidity.
**6.Monika et al., 2020** [9]	Clinical study	15	NR	NR	Excluded	15	NR	Maxillary molars	class III & IV Miller	PBPF	None	6 m	PBPF can be used in the treatment of class III and IV GR with reliable outcomes regarding RD, CAL, and PD.
**7.Kamal, 2021** [35]	Clinical study	40	20–55	NR	Excluded	20	20	Anterior & premolars	class II Miller	VISTA & NPBPF	VISTA & PRF	3,6 m	Both the PRF membrane & NPBPF are effective in Miller class II treatment using the VISTA with better root coverage obtained in the PRF group at 3, and 6 months.

The database search yielded thirty articles related to the treatment of GR by BPF. After eliminating duplicates through an automated process, twelve articles underwent screening by three independent reviewers, namely R.A, W.S, and H.E, to assess their eligibility. Five articles were excluded for not meeting the inclusion criteria. Some were case reports, and others were case series with fewer than ten patients. Finally, seven articles [9, 27, 28, 33–36] met the inclusion criteria and included in this review (Fig. 1). They included investigations involving six clinical studies, and only one case series with a sample size of ten participants (Table 1). Moreover, the data extracted from the involved studies included the number of recession defects, recession site, the class of GR, surgical techniques used, and the follow-up duration (Table 1).

### Risk of bias assessment

**Fig. 2:**
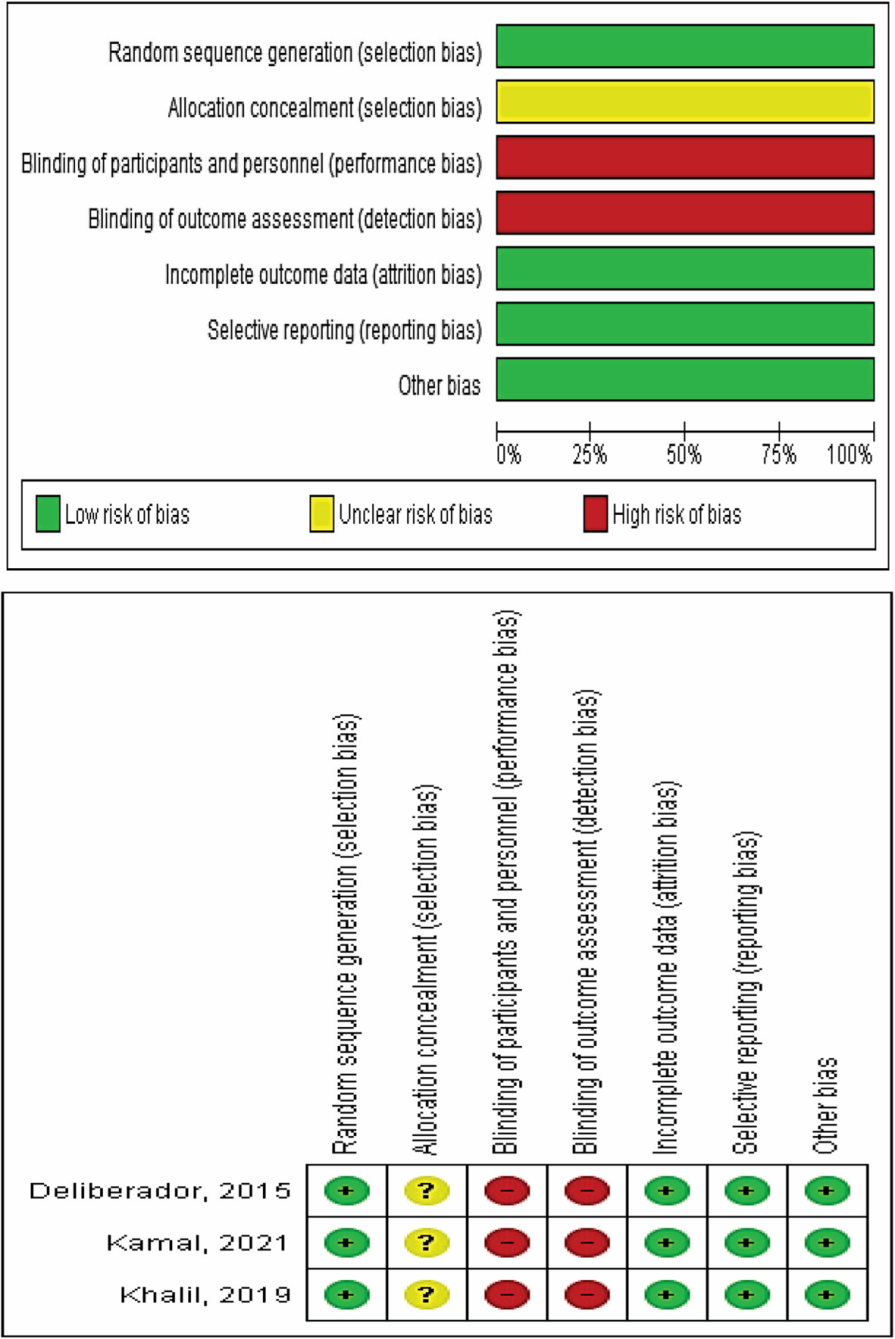
Risk of bias graph and summary

The risk of bias in the included studies was initially assessed by two independent reviewers (R.A and W.S) and then further reviewed by a third reviewer (H.E) using the Cochrane tool [37]. Only the RCTs were assessed based on the following domains: Random sequence generation, allocation concealment, blinding of participants and personnel, blinding of outcome assessments, incomplete outcome data, and selective reporting. The included RCTs were classified as low risk of bias if all domains were at low risk, unclear risk of bias was recorded if one or less of the domains were at unclear risk, and the RCTs were considered as high risk of bias if one or more domains were at high risk. However, if two or more domains were unclear, a medium risk of bias was assigned to the RCTs. The risk of bias graph and summary of the included RCTs are shown in (Fig. 2).

For non-randomized trials, we used appropriate risk of bias assessment tools which is cochrane risk of bias in non-randomized studies of interventions (ROBINS-I tool), and for single arm trials, we utilized the National Heart, Lung, and Blood Institute (NHLBI) Study Quality Assessment Tools. The risk of bias assessment for the rest of the included studies which were three single arm trials, and one non randomized controlled clinical trial provided in (Tables 2 and 3 respectively).

**Table 2:** Quality assessment for single arm trials based on the National Heart, Lung, and Blood Institute (NHLBI) study quality assessment tools

Criteria /study	Deepa & Kumar 2018	Kablan, 2018	Monika, 2020
1. Was the study question or objective clearly stated?	Yes	Yes	Yes
2. Were eligibility/selection criteria for the study population prespecified and clearly described?	Yes	No	Yes
3. Were the participants in the study representative of those who would be eligible for the test/service/intervention in the general or clinical population of interest?	Yes	Yes	Yes
4. Were all eligible participants that met the prespecified entry criteria enrolled?	Yes	Yes	Yes
5. Was the sample size sufficiently large to provide confidence in the findings?	No	Yes	Yes
6. Was the test/service/intervention clearly described and delivered consistently across the study population?	Yes	Yes	Yes
7. Were the outcome measures prespecified, clearly defined, valid, reliable, and assessed consistently across all study participants?	Yes	No	Yes
8. Were the people assessing the outcomes blinded to the participants’ exposures/interventions?	Not reported	Not reported	Not reported
9. Was the loss to follow-up after baseline 20% or less? Were those lost to follow-up accounted for in the analysis?	No	No	No
10. Did the statistical methods examine changes in outcome measures from before to after the intervention? Were statistical tests done that provided p values for the pre-to-post changes?	Yes	No	Yes
11. Were outcome measures of interest taken multiple times before the intervention and multiple times after the intervention (i.e., did they use an interrupted time-series design)?	Not reported	Not reported	Not reported
12. If the intervention was conducted at a group level (e.g., a whole hospital, a community, etc.) did the statistical analysis take into account the use of individual-level data to determine effects at the group level?	Not applicable	Not applicable	Not applicable

**Table 3:**
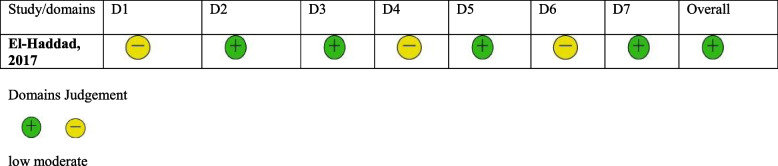
Risk of bias in non-randomized studies of interventions (ROBINS-I tool)

D1; bias due to confounding

D2; bias due to selection of participants

D3; bias in classification of interventions

D4; bias due to deviations from intended interventions

D5; bias due to missing data

D6; bias in measurement of outcomes

D7; bias in selection of the reported result

### Search outcomes

Several clinical measurements have been extracted from the included studies comprising the following parameters: RD, RW, WKG, PD, CAL, %RC, and TKG (shown in Table 4). We focused on the recorded measurements at both baseline and after 6 months follow-up duration. Furthermore, these outcomes were classified as primary outcomes which included RD, RW, WKG, and %RC while secondary outcomes included PD, CAL, and TKG.

**Table 4:** Clinical parameters of included studies

Study ID	Groups	RD (Mean ± SD)		RW (Mean ± SD)		WKG (Mean ± SD)		PD (Mean ± SD)		CAL (Mean ± SD)		%RC or (Mean ± SD) RC	TKG (Mean ± SD)	
		Baseline	Final (6 m)	Baseline	Final (6 m)	Baseline	Final (6 m)	Baseline	Final (6 m)	Baseline	Final (6 m)	Final (6 m)	Baseline	Final (6 m)
**1. Deliberador et al., 2015** [27]	**Group I** (BPF)	3.46 ± 1.03	1.25 ± 1.48	NR	NR	3.50 ± 1.24	5.08 ± 1.24	1.33 ± 0.49	1.50 ± 0.64	4.67 ± 1.07	2.75 ± 1.34	67.50%	1.08 ± 0.29	1.46 ± 0.45
	**Group II** (Control)	3.25 ± 1.14	1.08 ± 1.38	NR	NR	3.67 ± 1.61	5.42 ± 1.56	1.25 ± 0.45	1.67 ± 0.62	4.63 ± 1.19	2.83 ± 1.54	87.50%	1.08 ± 0.29	1.67 ± 0.65
**2. El-Haddad, 2017** [33]	**Group I** (BPF)	7.33± 0.91	3.53± 0.83	NR	NR	0.6± 0.49	5.4± 0.64	1.2± 0.55	1.93± 0.4	8.53± 1.12	4.86± 0.5	51.84%	NR	NR
	**Group II** (Control)	6.53± 0.5	2.99± 0.4	NR	NR	1.00± 0.62	4.99± 1.00	1.73± 0.76	1.86± 0.5	8.26± 1.06	4.86± 0.5	54.21%	NR	NR
**3. Deepa & Kumar 2018** [34]	**BPF**	6.4 ± 1.075	0.7 ± 0.6750	4.65 ± 0.4327	0.94 ± 1.350	0.6 ± 0.4	1.2 ± 0.42	1.9± 0.5676	1.1± 0.3162	NR	NR	89.30%	NR	
**4. Kablan, 2018** [28]	Group (BPF)	4.8 ± 1.8	1.7 ± 0.9	NR	NR	NR	NR	NR	NR	NR	NR	NR	NR	NR
**5. Khalil, 2019** [36]	**Group I** (BPF)	3.47± 0.50	1.04± 0.37	NR	NR	NR	NR	NR	NR	4.01± 0.39	1.90± 0.53	NR	NR	NR
	**Group II** (Control)	3.21± 0.61	0.86± 0.26	NR	NR	NR	NR	NR	NR	3.66± 1.01	1.96± 0.36	NR	NR	NR
**6. Monika et al., 2020** [9]	**Group** **BPF**	5.60± 1.18	2.87± 0.74	NR	NR	NR	NR	0.73± 0.59	1.73± 0.70	6.40± 1.18	4.53± 0.83	46.78%	NR	NR
**7. Kamal, 2021** [35]	**Group I** (BPF)	NR	1.45± 1.67	NR	NR	NR	5.20± 0.89	NR	1.70± 0.57	NR	2.45± 2.11	51.25± 36.53	NR	1.8± 0.37
	**Group II** (Control)	NR	0.80± 1.28	NR	NR	NR	5.55± 0.99	NR	1.85± 0.37	NR	1.25± 1.97	82.00± 30.22	NR	1.77± 0.25

*NR *not reported

## Results

The resulting systematic review pooled data from seven studies [9, 27, 28, 33–36]comprising of data from 117 patients with 136 GR defects. The age of participants ranges from (20 to 65) years old. The gender distribution among the studied population was 14 males and 18 females while 4 studies did not report the gender of participants. Different classes of Miller’s classification of GR were included (Class I & II & III & IV).

Regarding RD, Deepa and Kumar, 2018 [34] reported a statistically significant decrease from (6.4 ± 1.075) mm at baseline to (0.7 ± 0.675) mm at 6 months using the PBPF. In addition, El-Haddad and El-Shall’s study (2017) [33] revealed a reduction from (7.33 ± 0.91) to (3.53 ± 0.83) mm after 6 months in the PBPF group, compared to the PBPF with Emdogain group, where RD decreased from (6.53 ± 0.5) to (2.99 ± 0.4) mm.

Furthermore, Monika et al., 2020 [9] reported a statistically significant decrease in RD from (5.60 ± 1.18) to (2.87 ± 0.74) mm after 6 months in the PBPF group. Deliberador et al. in 2015 [27] demonstrated a significant reduction in RD from (3.46 ± 1.03) to (1.25 ± 1.48) mm after 6 months in the NPBPF group, whereas the SCTG group exhibited a significant decline from (3.25 ± 1.14) to (1.08 ± 1.38) mm after 6 months. Additionally, Khalil’s findings in 2019 [36] demonstrated a reduction in RD from (3.47 ± 0.50) to (1.04 ± 0.37) mm at the 6 months in the NPBPF group, whereas the SCTG group demonstrated a decrease from (3.21 ± 0.61) to (0.86 ± 0.26) after the same period (Table 4).

Interestingly, the higher percentage of root coverage (%RC) at 6- month follow-up reported in the PBPF group was (89.30%) according to Deepa & Kumar, 2018 [34] while the lowest (%RC) at 6 months in the same group was (46.78%) as shown by Monika et al., 2020 [9]. Additionally, El-Haddad and El-Shall’s study, 2017 [33] showed (51.84%) of RC at 6 months follow-up in the PBPF group while in the control group was (54.21%). Moreover, Deliberador et al., 2015 [27] showed (67.50%) of RC after 6 months in the NPBPF group while exhibiting a notably higher %RC of 87.50% in the control group. Furthermore, Kamal, 2021 [35] demonstrated a significant %RC at 6 months follow-up in the NPBPF (51.25 ± 36.53) compared to the control group (82.00 ± 30.22). All recorded clinical parameters of the included studies are shown in (Table 4).

The study by Deepa and Kumar in 2018 [34] indicates a noticeable increase in the WKG in the BPF group from baseline to 6 months after therapy. The mean values suggest a significant change, reflecting potential positive outcomes. In Deliberador et al., 2015 [27] study, both Group I (BPF) and Group II (Control) experienced an increase in WKG from baseline to 6 months follow-up after surgery. The BPF group exhibited a substantial improvement in WKG, and while the control group also showed lesser improvement, the difference between groups suggests a potential positive effect of the BPF treatment. Moreover, El-Haddad and El-Shall’s study in 2017 [33] reveals a significant increase in WKG for both the BPF and control (PBPF & Emdogain) groups. The BPF group’s WKG values indicate a notable improvement, suggesting a positive impact on keratinized gingiva width compared to the control group.

## Discussion

The current systematic review investigates the efficacy of BPF as a novel approach in the treatment of GR aiming to elucidate the underlying mechanisms driving the observed clinical outcomes. The present systematic review has incorporated data from 7 articles including 117 patients with 136 GR defects of different grades of Miller’s classification. The inclusion of different classes of GR according to Miller’s classification provides a comprehensive illustration of the efficacy of PBF in varying severities of GRs. The age and gender distribution of participants, encompassing a range of Miller’s classification classes, lay the foundation for understanding the diverse patient population examined in the included studies. Such diversity allows for a broader applicability of BPF in addressing GR across different demographic groups.

RD was consistently and statistically significantly reduced in all the studies included in this systematic review. The results of studies by Deepa and Kumar (2018) [34], El-Haddad and El-Shall (2017) [33], Khalil (2019) [36], Deliberador et al., (2015) [27], Monika et al., (2020) [9], and others all indicate a significant reduction in RD, suggesting that BPF may be a viable option in treating GR. The significant reduction in RD observed across various studies following BPF intervention reflects the regenerative potential of adipose tissue in promoting tissue healing and regeneration. Notably, BPF offers a rich source of adipose-derived stem cells (ADSCs) and growth factors, which play crucial roles in tissue repair processes [38, 39]. These bioactive components stimulate angiogenesis, fibroblast proliferation, extracellular matrix synthesis, and ultimately facilitating tissue regeneration and wound closure [40].

The analysis of RC% showed that different studies produced different outcomes. Monika et al. (2020) [9] recorded the lowest percentage of (%RC) at 46.78%, while Deepa and Kumar (2018) [34] reported an amazing 89.30% of RC in the PBPF group. Further information was supplied by Kamal (2021) [35] and El-Haddad and El-Shall (2017) [33], who compared the %RC between the PBPF and control groups. These variances highlight how crucial it is to consider various patient demographics and research designs when interpreting the results of %RC.

The wide range of %RC outcomes, spanning from 46.78 to 89.30% at 6 months, highlights the diversity in treatment outcomes observed across different study settings and methodologies. This variability could be attributed to several factors, including variations in surgical techniques, patient characteristics, follow-up durations, and measurement methods adopted in each study. As regards the GR treatment, three studies utilized PBPF in severe GR treatment of the upper molars with Miller’s Class III and IV [9, 33, 34], while four studies used the FBPF in class I and II GR treatment [27, 28, 35, 36].

The systematic review consistently underscored positive outcomes in the WKG following BPF treatment. Research by El-Haddad and El-Shall (2017) [33], Deliberador et al. (2015) [27], and Deepa and Kumar (2018) [34]demonstrated a significant rise in WKG in the BPF groups from baseline to the 6-month follow-up. This improvement points to a possible benefit of BPF for increasing the WKG. Most importantly, the variation in WKG improvement shown in El-Haddad and El-Shall’s study (2017) [33]between the BPF and control groups underlines the special role that BPF treatment has in fostering positive results .

The observed increase in WKG following BPF treatment highlights its potential to augment gingival tissues and improve periodontal health outcomes. The expansion of keratinized gingiva offers several clinical benefits, including enhanced gingival stability, reduced susceptibility to trauma, and improved esthetics [41].

Previously, BPF was used in several intraoral surgical procedures such as the closure of oroantral communications, closure of primary clefts or post-osteotomy clefts, and closure of post-excision maxillary defects as a result of benign and/or malignant tumors [17, 42–45]. Moreover, the BPF was used after ablative surgery or after fibrotic band incision in oral submucous fibrosis for coverage of mucosal defects. In addition, it was used as a membrane in sinus lift procedures and TMJ surgeries [46]. Recently, miscellaneous uses of BPF have been suggested such as vocal cord augmentation, in which the autologous fat harvested from the BPF is being injected intra-cordal [47]. Interestingly, PBFP has provided a considerable amount of keratinized tissue in the coverage of severe gingival recession defects in the upper molar teeth [22]. Furthermore, BPF also has been used in the treatment of Miller class I and II GR combined with different flap approaches such as VISTA and CAF [27, 28, 35].

Consequently, BPF graft usage in the GR surgical treatment has gained popularity in the last years as it can be used as pedicled (PBPF) in the upper molar area and free or non-pedicled (NPBPF) that can be used anywhere in any recession-type defect. As previously mentioned, BPF can be used in combination with different approaches either CAF or VISTA [9, 22, 28, 33, 35, 36]. The results of BPF in GR treatment are promising owing to their special characteristics as it is not subjected to lipolysis compared to other body fats [14, 46], high vascularity especially when it is used as a pedicled graft [14]. Moreover, it has the potential to epithelize with a slight contraction of the wound by 3 weeks after the surgical procedures [25, 26].

Furthermore, the minimal donor site morbidity associated with BPF harvesting underscores its advantages over traditional grafting techniques, such as SCTG. By avoiding the need for palatal tissue harvesting, BPF minimizes patient discomfort and accelerates postoperative recovery, thereby improving patient satisfaction and treatment outcomes [48].

Recently, BFP has been proved a source of stem cells which can be easily harvested from the oral cavity without causing further injury to the external body surface owing to its similar size between different people independent of both body weight and fat distribution all over the human body [49]. Buoyed by the positive outcomes, BPF emerges as a promising approach in the treatment of GR. Clinicians might consider the potential benefits of incorporating BPF into their treatment protocols, especially in cases where traditional methods may have several limitations [9, 22, 28, 33, 35, 36].

### Strengths of the review

This review represents the first systematic exploration of the use of BPF in the treatment of GR, filling a notable gap in the literature and providing valuable insights into this emerging treatment approach. Moreover, this review utilized a comprehensive search strategy across multiple electronic databases to include a diverse range of studies, such as RCTs, observational studies, and case series thus providing a broad scope of evidence for analysis. This review also analyzed various clinical parameters, including RD, %RC, WKG, PD, and CAL, providing a comprehensive evaluation of the effectiveness of BPF in GR treatment.

There are some limitations of the current systematic review including insufficient number of studies, deficient data, only English language studies included and shorter follow-up duration. Bias may be introduced by variations in patient demographics, study designs, and methodology. Furthermore, some studies did not report detailed methods of randomization selection of their cases that might affect the outcomes of their studies.

## Conclusion

Within limitations of this review, BPF can be considered as a promising graft to augment gingival tissues at different sites in the oral cavity with different Miller’s classes of GR providing a new era in GR treatment with minimal donor site morbidity, simple harvesting procedures, and easy manipulation of the harvested tissues. Several multicenter RCTs should be carried out, with larger sample sizes, and longer follow-up periods to provide a comprehensive insight regarding the use of BPF graft in GR therapy.

### Supplementary Information

Supplementary Material 1.

## Data Availability

The data that support the findings of this study are available from the corresponding author upon reasonable request.
